# GHSR suppression in neurons protects against aging-associated metabolic and cognitive impairments

**DOI:** 10.1007/s11357-025-01922-0

**Published:** 2025-10-11

**Authors:** Hongying Wang, Chia-Shan Wu, Danilo Landrock, Ariel Nevarez, Shanrun Liu, Anna Thalacker-Mercer, Yanfeng Zhang, Jamie I. Baum, Sunja Kim, Bingzhong Xue, Yuxiang Sun

**Affiliations:** 1 Department of Nutrition, Texas A&M University, College Station, TX 77843 USA; 2 Flow Cytometry and Single Cell Core Facility, University of Alabama at Birmingham, Birmingham, AL 35294 USA; 3 Department of Cell, Developmental and Integrative Biology, University of Alabama at Birmingham, Birmingham, AL 35294 USA; 4 Genetics Research Division, University of Alabama at Birmingham, Birmingham, AL 35294 USA; 5 Center for Human Nutrition, Department of Food Science, University of Arkansas System Division of Agriculture, Fayetteville, AR 72701 USA; 6 Texas A&M Preclinical Phenotyping Core, Texas A&M Institute for Genome Sciences and Society, Texas A&M University, College Station, TX 77843 USA; 7 Department of Biology, Georgia State University, Atlanta, GA 30303 USA

**Keywords:** Aging, GHSR, Obesity, Metabolism, Thermogenesis, Cognition, Inflammation

## Abstract

Aging is accompanied by progressive declines in metabolic and cognitive functions. Growth hormone secretagogue receptor (GHSR), a receptor for the gut hormone ghrelin, is highly expressed in neurons and plays a crucial role in metabolic regulation. We previously reported that aged global GHSR-ablated mice are lean and insulin-sensitive, and that neuronal GHSR-deleted mice (*Syn1‐cre;Ghsr*^*f/f*^) completely prevent diet‐induced obesity. However, the role of neuronal GHSR in metabolic and cognitive aging has not been elucidated. The current study aims to determine the roles of neuronal GHSR in aging metabolism and cognitive dysfunction. *Syn1‐cre;Ghsr*^*f/f*^ mice were subjected to cold stress, glucose- and insulin-tolerance tests, behavioral tests, and tissue analysis. Aging is accompanied by glycemic dysregulation and insulin resistance; old *Syn1‐cre;Ghsr*^*f/f*^ mice showed improved glucose tolerance and insulin sensitivity. Aging is associated with thermogenic impairment and cognitive decline; old *Syn1‐cre;Ghsr*^*f/f*^ mice showed better cold resistance and retained better recognition memory. Noticeably, there were increased expression of thermogenic makers (PGC1α and UCP1) and elevated sympathetic innervation markers (tyrosine hydroxylase and synaptophysin) in brown adipose tissue of old *Syn1‐cre;Ghsr*^*f/f*^ mice. Lastly, old *Syn1‐cre;Ghsr*^*f/f*^ mice exhibited decreased pro-inflammatory cytokines and increased neural plasticity-related markers (brain-derived neurotrophic factor, synaptophysin, and tyrosine hydroxylase) in metabolic and cognitive-relevant brain regions such as hypothalamus, cortex, and hippocampus. In conclusion, neuronal inhibition of GHSR promotes a healthy aging phenotype showing improved energy metabolism and cognitive function, which is likely contributed to the improved thermogenesis and insulin sensitivity, reduced inflammation, and restored neuronal plasticity.

**Supplementary Information:**

The online version contains supplementary material available at https://doi.org/10.1007/s11357-025-01922-0.

## Introduction

Aging is accompanied by increased risks of various diseases, impacting nearly every aspect of functionality, including physical, psychological, and cognitive functions [1]. Aging is commonly associated with obesity, which is caused by the imbalance of energy consumption and energy expenditure [2]. Another hallmark of aging is insulin resistance; controlling body weight has been shown to improve insulin sensitivity and glucose homeostasis in aging [3]. Insulin resistance is commonly associated with chronic inflammation prevalent in aging, coined as “inflammaging”; neuroinflammation has been linked to cognitive dysfunction [4, 5]. It has been shown that aging elevates pro-inflammatory cytokines in white adipose tissue, which disturb insulin signaling and impair glucose tolerance [6]. Additionally, aging is often accompanied by behavioral impairments, including cognitive dysfunction [7, 8], that can further lead to Alzheimer’s disease and dementia [9]. Aging not only affects white adipose tissue in storing energy, but also affects brown adipose tissue (BAT) in burning energy. We and others have shown that there is impaired thermogenesis in aging BAT [10–12]. However, the mechanisms underpinning aging-associated metabolic and cognitive impairments, and the interrelationship between metabolic dysfunction, inflammation, and neuronal functional decline in aging, remain poorly understood.

Ghrelin, often referred to as the “hunger hormone,” is a 28-amino acid peptide hormone secreted by the stomach that plays a key role in nutrient-sensing and energy homeostasis [13, 14]. Growth hormone secretagogue receptor (GHSR) is the functional receptor of ghrelin and is highly expressed in neurons [15, 16]. It is known that ghrelin can easily cross the blood–brain barrier and bind to the ghrelin receptor GHSR in the brain [17]. Our study showed that global ablation of GHSR has significant impacts on metabolism and protects against age-associated obesity, insulin resistance, and inflammation [11, 18]. It has been reported that ghrelin levels are negatively correlated with body weight and insulin resistance [19]. The insulin action in the brain has been linked to cognitive functions [20]. Ghrelin and GHSR play multifaceted roles in the brain, regulating appetite, thermogenesis, and glucose-sensing, etc. [21]. Elevated GHSR level in the hippocampus by virus injection impairs memory encoding and contributes to AD-associated memory deficits [22]. To specifically study GHSR in neurons, we have generated a neuron-specific GHSR ablation mouse model (*Syn1‐cre;Ghsr*^*f/f*^). We previously reported that neuronal deletion of GHSR protects against high fat diet-induced obesity and insulin resistance in mice by increasing energy expenditure [23], and mitigates diet-induced depression and memory impairment via AMPK-autophagy signaling-mediated neuroinflammation [15]. However, it is unknown whether neuronal GHSR affects metabolic and cognitive functions in aging. In this study, we have further investigated the role and mechanism of neuronal GHSR in aging-associated metabolic and cognitive impairments.

## Materials and methods

### Animals

As we previously reported, to generate neuronal GHSR-deficient mice, we used *synapsin 1-Cre* (*Syn1‐cre*) mice to breed with *Ghsr*^*f/f*^ to obtain the *Syn1‐cre;Ghsr*^*f/f*^ mice [23]. Age-matched male *Ghsr*^*f/f*^ and *Syn1‐cre;Ghsr*^*f/f*^ mice were fed with a regular diet (RD) from Harlan Teklad 2020X (Madison, WI, USA). Mice were housed in the Texas A&M University (College Station, Texas, USA) animal facility with 12 h of alternating light/dark cycles at 23 °C ± 1 °C ambient temperature. The composition of the RD was 16% calories from fat, 60% calories from carbohydrates, and 24% calories from protein. Young (4–6 months old) and old (20–22 months old) male mice were used for the experiments. Body weights were measured monthly. All experimental protocols were approved by the Institutional Animal Care and Use Committee (IACUC) of Texas A&M University (College Station, Texas, USA).

### Glucose tolerance test (GTT)

As previously reported [24], mice were fasted overnight for 16 h before conducting GTT; we measured body weights on the day of the test. We injected d-glucose (2.0 g/kg of body weight) by intraperitoneal injection based on each mouse’s body weight. Blood glucose was measured by a glucometer, and 25 μL of blood was collected from the tail veins in EDTA-coated tubes at the following time points: 0, 15, 30, 60, and 120 min. Plasma was isolated and stored at − 80 °C for insulin measurement.

### Insulin tolerance test (ITT)

As reported [25, 26], the mice were fasted for 6 h starting at 8:00 AM. Insulin solution (0.1 units/mL) was diluted from Humulin (100 units/mL) with saline. Insulin (1 unit/kg of body weight) was intraperitoneally injected based on the body weight of each mouse. Blood glucose was measured at the points 0, 30, 60, and 120 min after insulin injection. If blood glucose dropped below 40 mg/dL at any time point, 300 ml of 20% dextrose was *i.p.* injected to rescue the mouse from hypoglycemia.

### Enzyme-linked immunosorbent assay for serum insulin

As previously reported [27]. Serum insulin levels were measured using an enzyme-linked immunosorbent assay (ELISA) kit for insulin, following the instructions from the manufacturer (Mercodia, Uppsala, Sweden).

### Quantitative real-time PCR

As we reported [28, 29], total RNA was extracted using an Aurum™ Total RNA Mini Kit (Bio-Rad, CA, USA). cDNA was synthesized from total RNA using the iScript™ Reverse Transcription Supermix (Bio-Rad, CA, USA). Quantitative real-time PCR was performed in duplicates using the SsoAdvanced™ Universal SYBR® Green Supermix (Bio-Rad, CA, USA) on the CFX384 Touch™ Real-Time PCR Detection System (Bio-Rad, CA, USA). Relative mRNA expression levels were normalized by the housekeeping gene, cyclophilin. Primer information is provided in Table 1 below.

**Table 1:** Sequences of primers (5′- > 3′) used in the quantitative RT-PCR analysis

mRNA	Forward	Reverse
IL-1β	TGTTCTTTGAAGTTGACGGACCC	TCATCTCGGAGCCTGTAGTGC
IL-6	CCAGAGATACAAAGAAATGATGG	ACTCCAGAAGACCAGAGGAAAT
UCP1	GTGAAGGTCAGAATGCAAGC	AGGGCCCCCTTCATGAGGTC
BDNF	GGGTCACAGCGGCAGATAAA	GCCTTTGGATACCGGGACTT
Cyclophilin	GCTGGACCAAACACAAACGG	ATGCTTGCCATCCAGCCATT

### Cold stress study

The cold stress study was adapted from our previous report [30]. Briefly, core body temperatures in mice were measured using a TH-8 Thermalert Clinical Monitoring Thermometer (Physitemp Instruments, Inc., NJ, USA). Mice were individually caged and exposed to 4 °C for 4–5 h. Body temperature was recorded hourly at 0, 1, 2, 3, 4, and 5 h. After the final measurement, the mice were immediately sacrificed.

### Fear conditioning test

This test was conducted as we previously described [31, 32] to evaluate memory function. Test chambers with clear Plexiglas sides and electrified grid floors were placed inside sound-attenuated enclosures. On the training day, mice were allowed to freely explore the chamber for 2 min with the lights on. A conditioned stimulus (CS) at 80 dB noise was presented for 30 s, followed by a 2-s mild foot shock of unconditioned stimulus (US) at 0.5 mA. A second and third CS-US pairing occurred after a 2-min interval. Mice were returned to their home cages. The FreezeFrame5 monitor system was used to control the stimulus timing and record freezing behavior. Mice were excluded from the test if they failed to show any response to the foot shock. After 24 h of training, contextual and cued auditory fear conditioning were assessed. For contextual fear testing, mice were reintroduced to the original chamber for 5 min, and the freezing behavior was recorded without additional stimuli. Two hours later, the auditory CS test was conducted in a modified chamber as described below to assess cue-dependent fear response. Mice were placed in a modified chamber with clear red plastic inserts, vanilla-scented odor, and no bedding to alter the environmental context. Testing involved two phases: a 3-min pre-CS phase without auditory stimuli, followed by a 3-min CS phase with the 80-dB tone. Freezing behavior during these phases was recorded, and the percentage of freezing time during the CS phase minus pre-CS freezing was calculated to determine the auditory fear response.

### Novel object recognition test

A novel object recognition test was conducted as previously described [33] to evaluate recognition memory. The novel object recognition test was conducted over three consecutive days, with distinct phases: acclimatization, training, and testing. On the first day, mice were placed individually in an open-field apparatus (40 × 40 × 50 cm) constructed with transparent plastic for a 5-min session without objects to allow habituation to the testing environment. Twenty-four hours after acclimatization, mice were introduced to the same apparatus containing two identical objects (A1 and A2) positioned in adjacent corners, each approximately 9 cm from the walls. Mice were then allowed to explore freely for 5 min. Two hours after the training session, mice were returned to the apparatus containing one familiar object (A1) and one novel object (B). Exploration was recorded during a 5-min session to assess the recognition memory. Object exploration, defined as sniffing or touching an object with the nose, was monitored and recorded using the ANY-maze tracking system (Stoelting, USA). Recognition memory was quantified using a recognition index calculated as: TA is the time spent exploring the familiar object, and TB is the time spent exploring the novel object. The recognition index (RI) is calculated by TB/(TA + TB). The RI index reflects the preference for the novel object, indicative of recognition memory performance.

### Open field test

An open field test was performed as previously described [15]. Mice were placed in the test room 1 h before the test. Each mouse was allowed to stay in a standardized chamber (50 cm length × 50 cm width × 38 cm height) for 30 min. The mice naturally like to stay in the corner/outer zone of the square; therefore, the travel distance, mobility time, and time spent in the outer/center zones were recorded and analyzed as an indication of physical activity and anxiety status.

### Immunofluorescence staining

As we previously reported [15, 34], the mouse brains were collected and immediately fixed in 10% formalin for 24 h at room temperature. The brains were then cryoprotected by immersion in 30% sucrose in PBS at 4 °C until sectioning. Coronal Sects. (30 µm) were obtained using a cryostat (Leica, Wetzlar, Germany). Floating sections were then immuno-stained with primary antibodies overnight and then with secondary antibodies for 2 h. A secondary antibody-only control was included each time as a negative control. The sections were mounted onto SuperFrost glass slides and cover-slipped using a Mounting Shield with DAPI (Vector Laboratories, CA, USA). The slides were stored in the dark at 4 °C, and the images were taken with a Leica confocal microscope (Leica TCS SPE, Wetzlar, Germany). Images were quantified by the ImageJ software; briefly, after opening the single-channel picture in ImageJ, set the image type to “8 bit,” then adjust the threshold to select the areas with positive fluorescent signal compared with the negative control, and measure the integrated density. Antibody information is as follows: Rabbit anti-tyrosine hydroxylase antibody (Cat# 2792S, Cell Signaling Technology, MA, USA); rabbit anti-BDNF antibody (Cat# PA5-85,730, Thermo Fisher Scientific, MA, USA); mouse anti-NeuN antibody (Cat# ab104224, abcam, MA, USA); rabbit anti-synaptophysin antibody (Cat# ab52636, abcam, MA, USA); goat anti-rabbit secondary antibody Alexa Fluor™ 488 (Cat# A-11008, Thermo Fisher Scientific, MA, USA); goat anti-mouse secondary antibody Alexa Fluor™ 594 (Cat# A-11005, Thermo Fisher Scientific, MA, USA).

### Western blotting

Western blotting was performed as previously described [15, 35]. Briefly, tissues were homogenized and centrifuged to get the protein supernatant. Protein samples were quantified, prepared in 6X reducing SDS sample buffer (Cat# AAJ61337AD, Thermo Fisher Scientific, MA, USA), and denatured at 99 °C for 5 min. Subsequently, protein samples (10 μg/sample) were separated by sodium dodecyl sulfate–polyacrylamide gel electrophoresis and then transferred to nitrocellulose membranes for immunoblot analysis. Membranes were incubated overnight at 4 °C with primary antibodies in a 5% BSA solution, followed by incubation with a secondary antibody in a 5% BSA solution the following day. Clarity Western ECL Substrate (Cat# 1,705,061, Bio-Rad, CA, USA) was used to incubate the membrane to generate light signals that were captured by the ChemiDoc™ Imaging System (Cat# 12,003,153, Bio-Rad, CA, USA). The protein band intensity was quantified by the ImageJ software. Antibody information is as follows: Rabbit anti-PGC1α antibody (Cat# 2178S, Cell Signaling Technology, MA, USA); rabbit anti-UCP1 antibody (Cat# ab10983, abcam, MA, USA); rabbit anti-synaptophysin antibody (Cat# ab52636, abcam, MA, USA); rabbit anti-tyrosine hydroxylase antibody (Cat# 2792S, Cell Signaling Technology, MA, USA); rabbit anti-GAPDH antibody (Cat# 2118L, Cell Signaling Technology, MA, USA); Horseradish peroxidase (HRP)-linked anti-rabbit IgG (Cat# 7074, Cell Signaling Technology, MA, USA).

### Statistical analysis

Data was analyzed using an unpaired two-tailed *t*-test, and one or two-way ANOVA with Tukey’s post hoc test by GraphPad Prism software. Results are presented as mean ± SEM, and *P* values < 0.05 were viewed as statistically significant.

## Results

### Age-dependent decline of thermogenic function

**Fig. 1:**
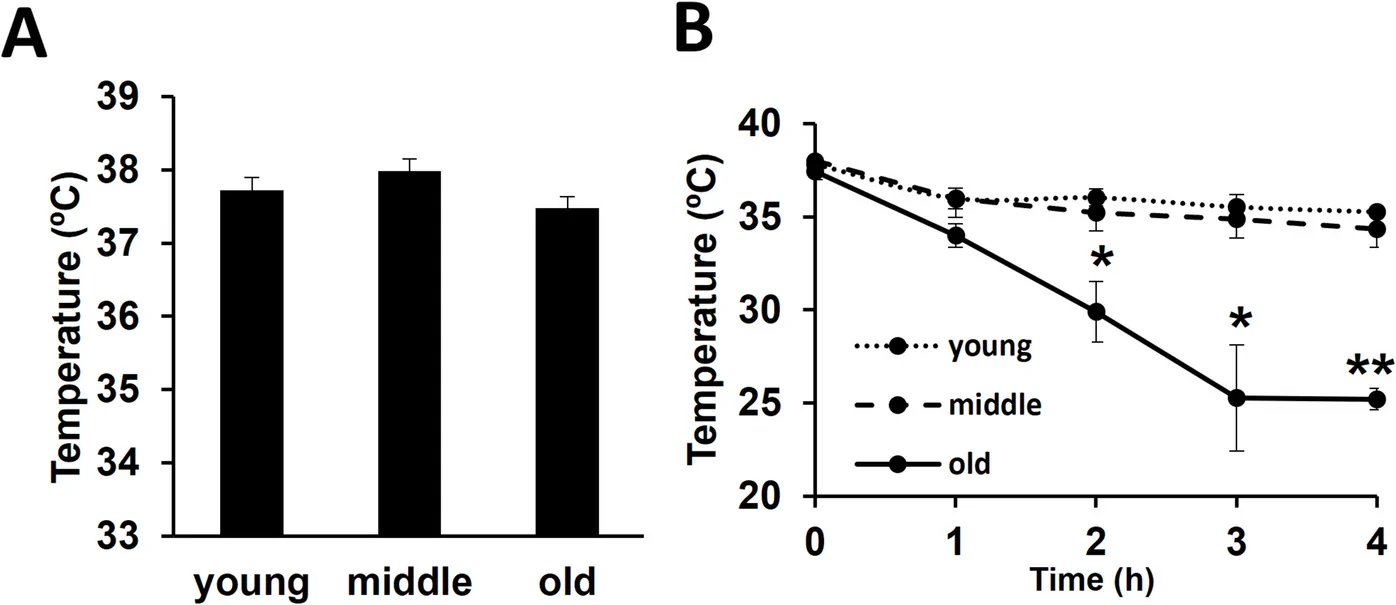
Age-associated decline of thermogenic function. **A** Core body temperature of mice at different ages. **B** Core body temperature of different-aged mice under 4 °C cold stress. *n* = 4 of young mice at 4–6 months old; *n* = 5 of middle-aged mice at 14 months old; *n* = 5 of old mice at 20–22 months old. *p < 0.05, **p < 0.01. One-way ANOVA with Tukey’s post hoc test was used. All data are presented as means ± SEM

Under normal room temperature (23 °C ± 1 °C), the core body temperature of all young, middle-aged, and old mice remained stable (Fig. 1A). Upon exposure to cold stress (4 °C), the body temperature of old mice began to drop dramatically after 2 h of exposure, while young and middle-aged groups experienced only moderate reduction of body temperature (Fig. 1B). These results indicate that aging significantly impairs cold-induced thermogenesis, old mice lost the ability to maintain body temperature in response to a cold challenge.

### Suppression of neuronal GHSR preserves thermogenic function and insulin sensitivity in aging

**Fig. 2:**
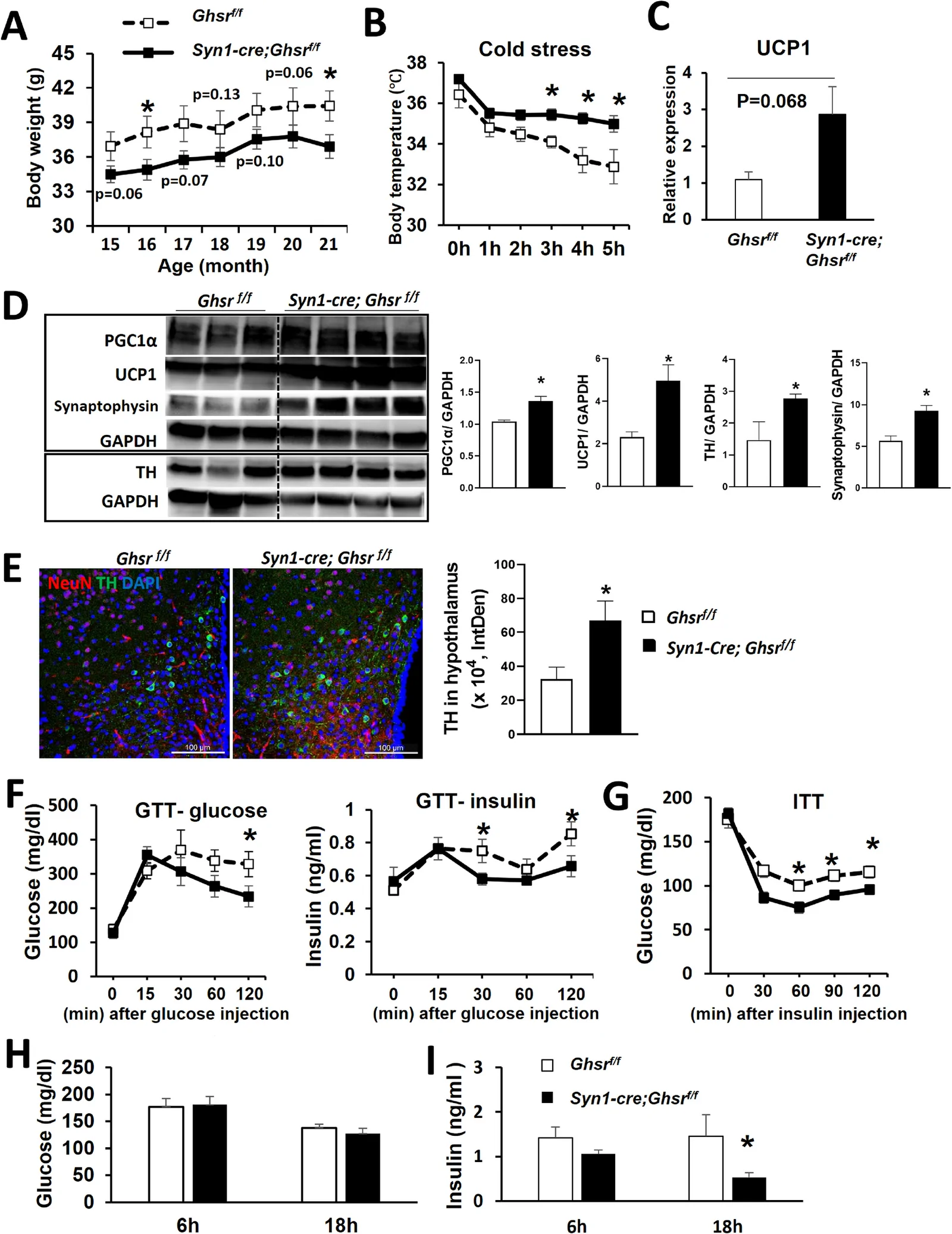
Suppression of neuronal GHSR preserves thermogenic function and improves insulin sensitivity in aging. Male *Syn1-cre;Ghsr*^*f/f*^ and *Ghsr*^*f/f*^ mice at 20–22 months old were used. **A** Body weights.** B** The curves of core body temperatures under cold stress (4℃). **C** UCP1 gene expression in inguinal adipose tissue. **D** Western blotting and subsequent quantification to determine relative protein expression in BAT. **E** Representative coronal sections of immunofluorescence staining and quantification of integrated density (IntDen) for TH in the hypothalamus of old mice. NeuN (red), TH (green), and nuclear counterstain (DAPI, blue). Mouse number *n* = 3, 3 sections/brain. Scale bar 100 µm. Under a 20 × objective lens. **F** Glucose (left) and insulin (right) levels during GTT. **G** Glucose levels during ITT. **H**,** I** Glucose and Insulin levels after 6-h and 18-h fasting. *n* = 6 *Ghsr*^*f/f*^ mice, and *n* = 9 *Syn1-cre;Ghsr*^*f/f*^ mice. *p < 0.05. An unpaired two-tailed *t*-test and two-way ANOVA with Tukey’s post hoc test were used to compare *Syn1-cre;Ghsr*^*f/f*^ vs. *Ghsr*^*f/f*^. All data are presented as means ± SEM

We previously found that there is no significant difference in thermogenic function and insulin sensitivity between *Syn1‐cre;Ghsr*^*f/f*^ and *Ghsr*^*f/f*^ control mice at a young age [23], so we mostly focused on old mice in this study. We found that the body weights showed a decreased trend in old *Syn1‐cre;Ghsrf/f* mice compared with old *Ghsr*^*f/f*^ mice, and reached statistical difference at 21 months old of age near termination (Fig. 2A). Under 4 °C cold exposure, old *Syn1‐cre;Ghsr*^*f/f*^ mice maintained body temperature much better than *Ghsr*^*f/f*^ mice after 5 h of cold stress, indicating that neuronal GHSR deficiency prevents aging-associated thermogenic impairment (Fig. 2B). Consistently, we found that the level of a thermogenic-related gene uncoupling protein 1 (UCP1) in inguinal fat showed an increased trend (*p* = 0.068) in *Syn1‐cre;Ghsr*^*f/f*^ mice compared with that of controls (Fig. 2C). Similarly, Western blotting data showed that old *Syn1‐cre;Ghsr*^*f/f*^ mice had significantly higher protein expression of PGC1α and UCP1 than the controls as well, signifying a stronger activation of a critical thermogenic program in generating heat to defend against hypothermia (Fig. 2D). In addition, we also assessed the sympathetic innervation markers of tyrosine hydroxylase (TH) and synaptophysin in BAT and found that both TH and synaptophysin were markedly increased in *Syn1‐cre;Ghsr*^*f/f*^ mice compared with *Ghsr*^*f/f*^ mice (Fig. 2D). It is well documented that hypothalamus in the brain is important for regulation of thermogenesis and glucose metabolism; indeed we found the TH levels, detected by the green fluorescence, were significantly higher in the hypothalamus of *Syn1‐cre;Ghsr*^*f/f*^ mice than that of controls, while the NeuN labeled neurons (Fig. 2E) and synaptophysin (Fig. S1) did not show difference between the two genotypes. These results together suggest that neuronal GHSR plays an important role in modulating thermogenesis in aging under cold stress.

To further determine the effect of neuronal GHSR on glucose homeostasis and insulin sensitivity in aging, we performed GTT and ITT. Old *Syn1‐cre;Ghsr*^*f/f*^ mice exhibited significantly reduced blood glucose levels in GTT, accompanied by lower insulin in *Syn1‐cre;Ghsr*^*f/f*^ mice (Fig. 2F). Old *Syn1‐cre;Ghsr*^*f/f*^ mice also exhibited significantly reduced blood glucose during ITT (Fig. 2G). Moreover, while there was no significant difference in blood glucose after 6 and 18 h fasting between old *Syn1‐cre;Ghsr*^*f/f*^ and controls (Fig. 2H), old *Syn1‐cre;Ghsr*^*f/f*^ mice exhibited lower blood insulin levels after 18 h of fasting (Fig. 2I). These data suggest that neuron-specific GHSR plays important roles in regulating glucose homeostasis and insulin sensitivity in aging.

### Suppression of neuronal GHSR ameliorates aging-associated decline in memory function

**Fig. 3:**
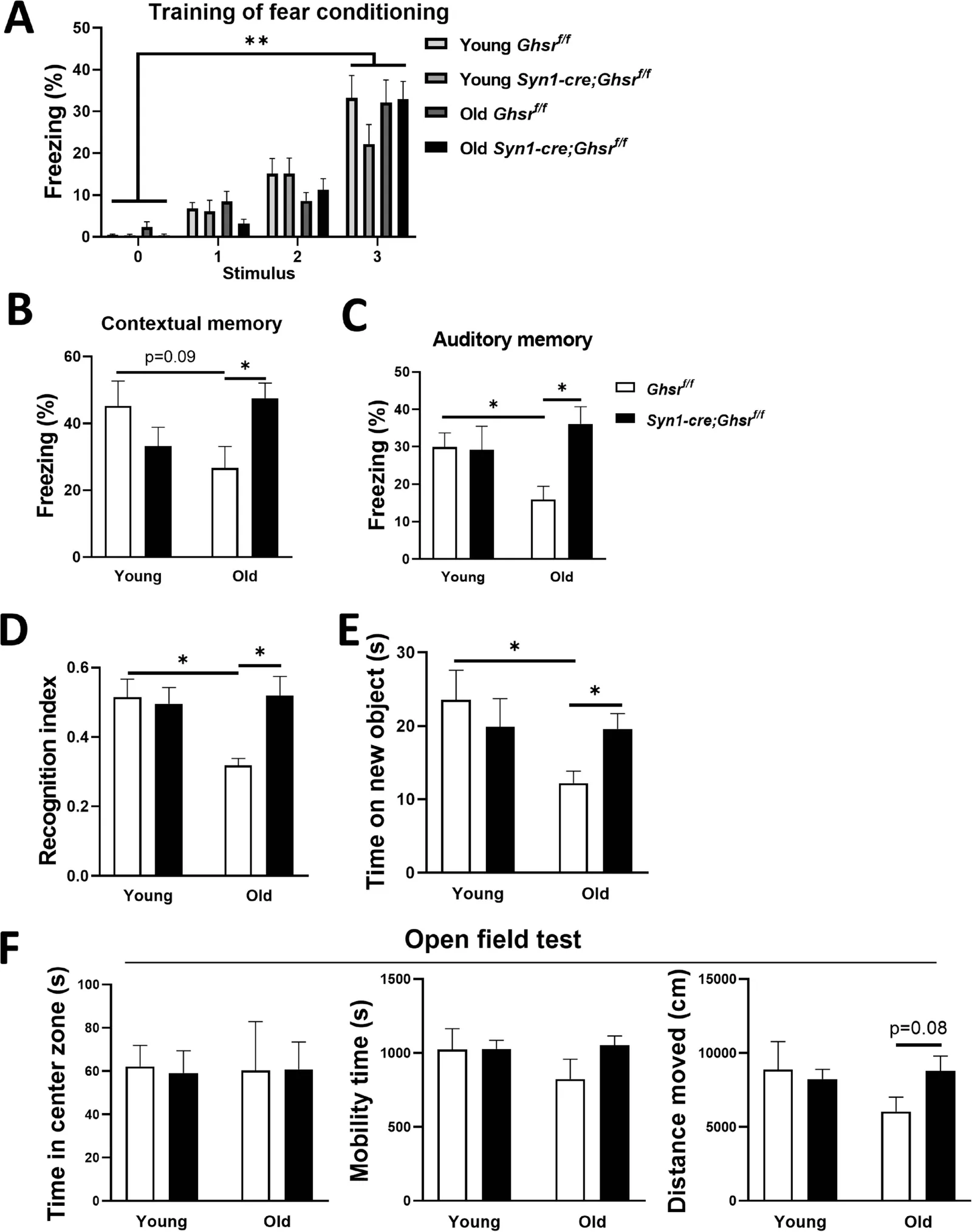
Suppression of neuronal GHSR protects against cognitive decline in aging. **A** Freezing score during training in the fear conditioning test. **B** Freezing score in the fear conditioning test to assess contextual memory. **C** Freezing score in the fear conditioning test to assess auditory memory.** D**, **E** Recognition ratio and time spent on the novel object in the novel object recognition test to assess the recognition memory. **F** Time in the center zone, mobility time, and distance moved were recorded in the open field test. Young mice were 4–6 months old; old mice were 20–22 months old. *n* = 6–9. *p < 0.05, **p < 0.01; two-way ANOVA with Tukey’s post hoc test was used. All data are presented as means ± SEM

To investigate whether neuronal GHSR inhibition affects learning and memory in aging, we conducted a fear conditioning test to assess conditioned fear memory. All mice responded to the sound and electric shock stimuli appropriately during the training phase, showing a gradually increased freezing score after each stimulus (Fig. 3A). After 24 h of training, mice were returned to the same chamber without any stimuli to assess their contextual memory. A trend of reduced freezing behavior (*p* = 0.09) was observed in old mice, in line with the known memory decline in aging. Remarkably, old *Syn1‐cre;Ghsr*^*f/f*^ mice exhibited a significantly higher freezing percentage than that of old control mice, indicating that neuronal GHSR deficiency improves contextual memory in aging (Fig. 3B). Next, the mice were placed in a different chamber with a new odor and were exposed to a sound stimulus to assess their auditory recall memory. Consistently, we observed that old *Ghsr*^*f/f*^ mice had a significantly lower freezing score compared to young mice, showing a decline of auditory memory, indicating the age-associated memory loss was attenuated in mice with neuronal GHSR deletion (Fig. 3C). There were no significant differences in fear conditioning between young *Ghsr*^*f/f*^ and *Syn1‐cre;Ghsr*^*f/f*^ mice (Fig. 3A–C).

In addition, we conducted a novel object recognition test to assess recognition memory. While there was no difference between genotypes in the young mice, we found that old *Ghsr*^*f/f*^ mice showed decreased recognition memory, demonstrated by the recognition index and exploration time of the new object, whereas old *Syn1‐cre;Ghsr*^*f/f*^ mice preserved recognition memory significantly better than old control mice (Fig. 3D, E). We also conducted an open field test to assess the anxiety status (Fig. 3F); we found there was no difference in terms of anxiety demonstrated by the time in the center zone, mobility time, and distance traveled during the test. Collectively, these results reveal that aging impairs cognition, and neuronal GHSR deletion improves aging-induced cognitive decline.

### Suppression of neuronal GHSR downregulates proinflammatory cytokines in the aging brain

**Fig. 4:**
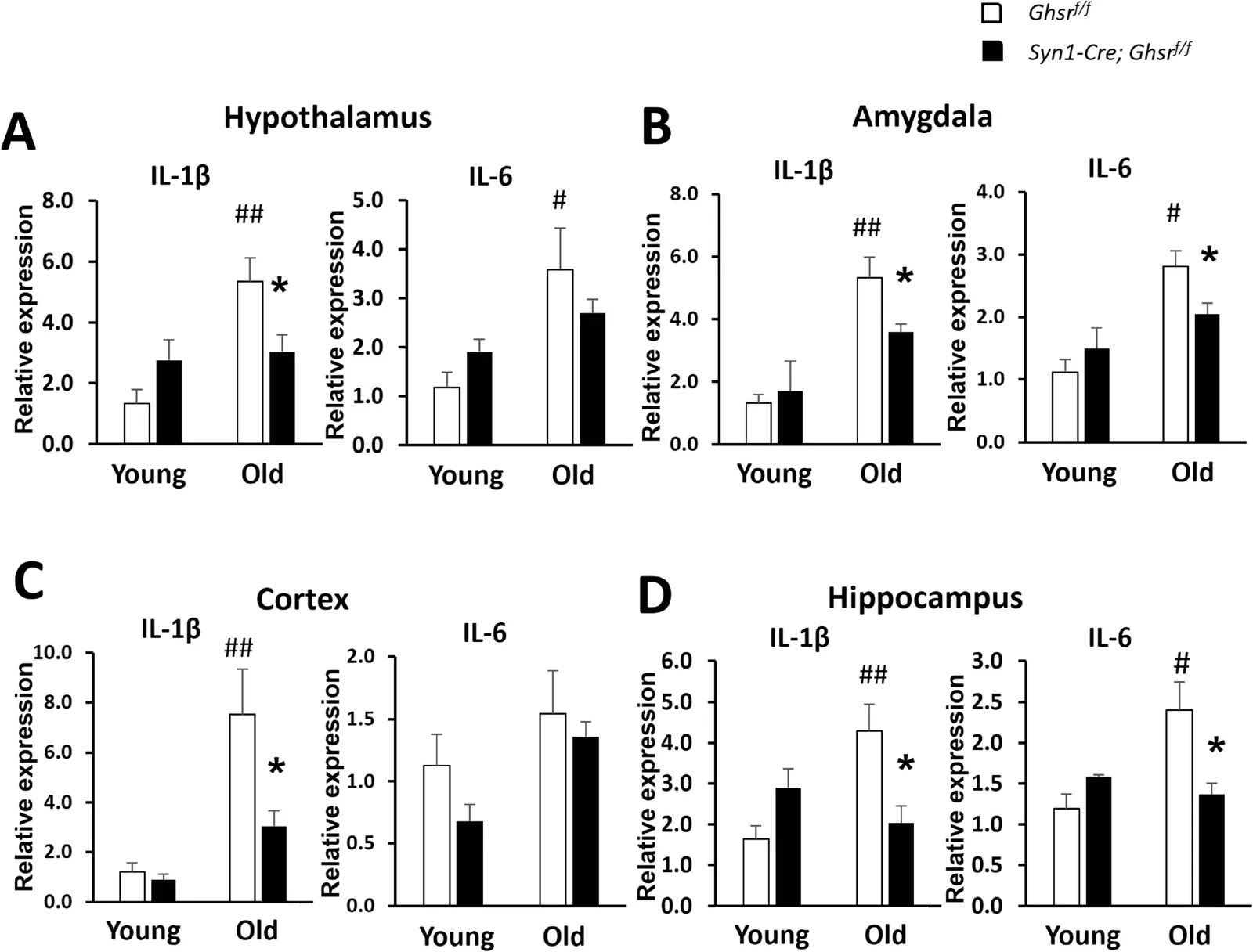
Suppression of neuronal GHSR decreases inflammation in the brain. Gene expressions of 4–6-month-old male mice (*n* = 4) and 20–22-month-old male mice (*n* = 7–8) in the hypothalamus (**A**), amygdala (**B**), cortex (**C**), and hippocampus (**D**). #*p* < 0.05, ##*p* < 0.01; old vs. young. *p < 0.05, *Syn1-cre;Ghsr*^*f/f*^* vs. Ghsr*^*f/f*^. Two-way ANOVA with Tukey’s post hoc test was used. All data are presented as means ± SEM

It is well known that aging is commonly associated with low-grade chronic inflammation throughout the body [5]. As expected in control mice, both IL-1β and IL-6 levels were significantly increased in brains of old mice compared to those of young mice, specifically in regions of hypothalamus, amygdala, cortex, and hippocampus. Intriguingly, brain IL-1β and IL-6 levels were remarkably lower in these regions of old *Syn1‐cre;Ghsr*^*f/f*^ mice compared to those of old control mice (Fig. 4A–D). These data demonstrate that neuronal deletion of GHSR mitigates inflammation in the aging brain.

### Suppression of neuronal GHSR upregulates neural plasticity-related markers in the aging brain

**Fig. 5:**
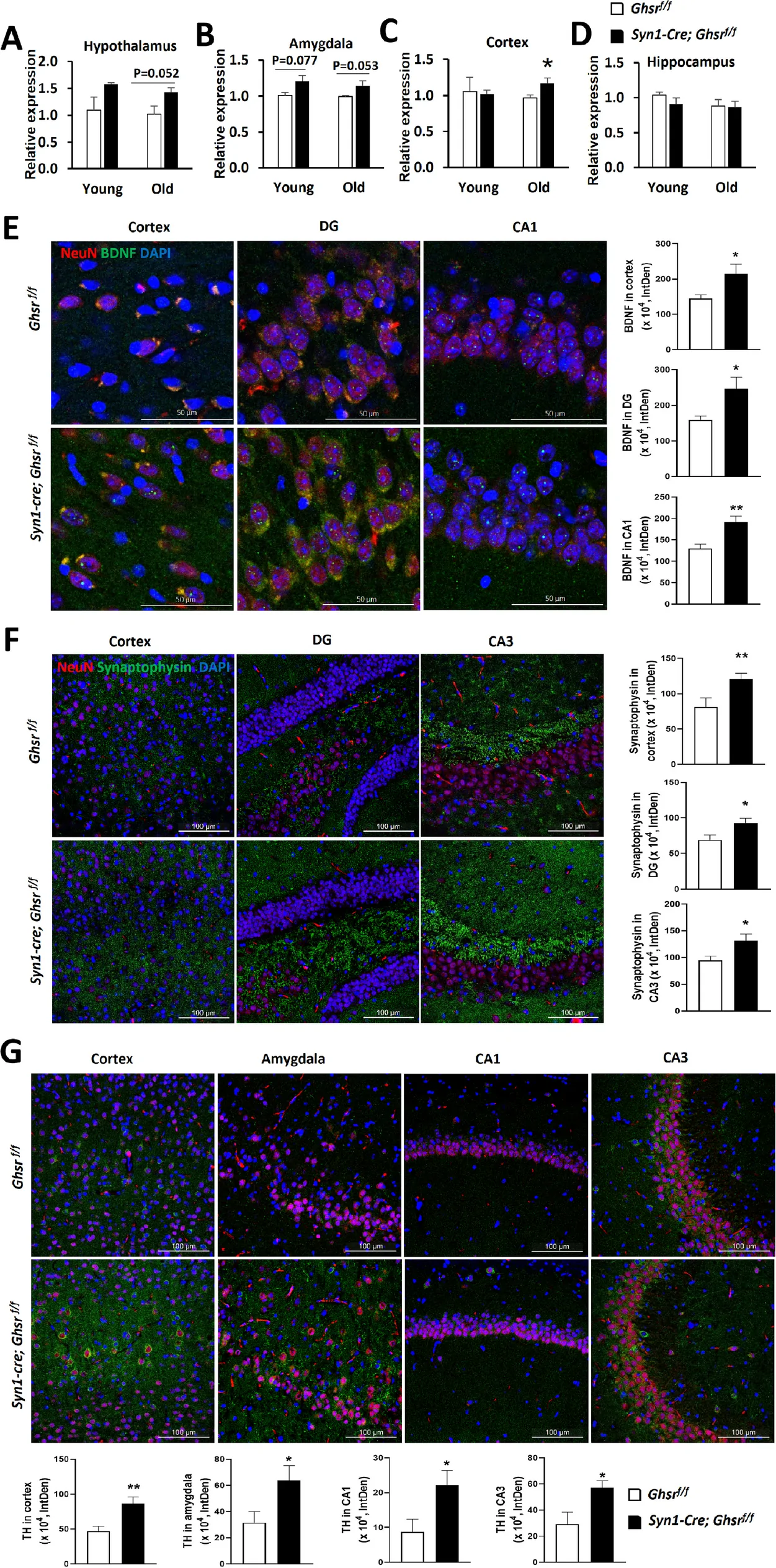
Suppression of neuronal GHSR elevates neural plasticity-related markers in the aging brain. BDNF gene expression of 4–6-month-old male mice (*n* = 4) and 20–22-month-old male mice (*n* = 7–8) in the hypothalamus (**A**), amygdala (**B**), cortex (**C**), and hippocampus (**D**). **E** Representative coronal sections of NeuN and BDNF immunofluorescence staining, and quantification of integrated density for BDNF in the cortex, hippocampal DG, and CA1 regions of the old mice. NeuN (red), BDNF (green), and nuclear counterstain (DAPI, blue); scale bar 50 µm. **F** Representative coronal sections of NeuN and synaptophysin immunofluorescence staining, and quantification of integrated density for synaptophysin in the cortex, hippocampal DG, and CA3 regions of the old mice. NeuN (red), synaptophysin (green), and nuclear counterstain (DAPI, blue); Scale bar 100 µm. **G** Representative coronal sections of NeuN and TH immunofluorescence staining, and quantification of integrated density for TH in the cortex, hippocampal DG, CA1, and CA3 regions of the old mice; NeuN (red), TH (green), and nuclear counterstain (DAPI, blue); scale bar 100 µm. Mouse number *n* = 3, 3 sections/brain. Under a 20 × objective. *p < 0.05, **p < 0.01, *Syn1-cre;Ghsr*^*f/f*^* vs. Ghsr*^*f/f*^. Two-way ANOVA with Tukey’s post hoc test and an unpaired two-tailed *t*-test were used. All data are presented as means ± SEM

Brain-derived neurotrophic factor (BDNF) is a key factor that has a protective effect on synaptic plasticity, critical for cognitive function [36]. In old *Syn1‐cre;Ghsr*^*f/f*^ mice, BDNF mRNA levels showed a trend of increase in the hypothalamus (*p* = 0.052) and amygdala (*p* = 0.053), and a significant increase in the cortex (Fig. 5A–D). To further assess BDNF protein levels, we carried out immunofluorescence staining. There were significantly elevated BDNF protein levels detected in the cortex and hippocampal regions, including CA1 and dentate gyrus (DG), in old *Syn1‐cre;Ghsr*^*f/f*^ mice compared to controls (Fig. 5E), suggesting improved neural plasticity. We also found that the synaptophysin levels were increased in the cortex, DG, and CA3 areas of old *Syn1‐cre;Ghsr*^*f/f*^ mice (Fig. 5F), suggesting improved synapse density. In addition, we detected significantly more TH expressions in the cortex, amygdala, CA1, and CA3 of old *Syn1‐cre;Ghsr*^*f/f*^ mice than in controls (Fig. 5G), while no difference was detected in NeuN-labeled neurons (Fig. 5E–F). The negative controls of these immuno-staining are shown in Fig. S2. These data collectively demonstrate that the ablation of neuronal GHSR upregulates neural plasticity in the behavior-relevant brain regions of the cortex and hippocampus in aging.

## Discussion

In this study, we reported the role of neuronal GHSR in aging-associated metabolic and cognitive dysfunctions. Neuronal GHSR-deficient mice showed anti-aging effects across various aspects of aging, including glucose homeostasis, insulin resistance, thermogenesis, and cognitive function. We previously reported that global GHSR ablation improved insulin resistance, glucose homeostasis, and thermogenesis in aging [11, 12]. Our new data for old *Syn1‐cre;Ghsr*^*f/f*^ mice further demonstrate that these metabolic improvements are likely resulted primarily by GHSR inhibition in neurons. These neuronal GHSR-associated phenotypes are aging-dependent, as we previously reported; there were no differences in metabolism and cognition observed in young mice [15, 23]. The aging mice in the current study were fed a regular diet. We previously reported that neuronal GHSR deletion shows a protective effect in metabolism under diet-induced obesity [23]. The differential phenotypes of neuronal GHSR under different ages and metabolic states suggest that neuronal GHSR functions as a nutrient-sensor, reflecting the body’s biological conditions.

We observed an impressive improvement in insulin sensitivity in old *Syn1‐cre;Ghsr*^*f/f*^ mice, which is likely due to improved non-shivering thermogenesis. We found that old *Syn1‐cre;Ghsr*^*f/f*^ mice preserved body temperature better, consistently showing significantly higher levels of PGC1α and UCP1 protein in BAT under cold stress. Furthermore, we identify a potential mechanism for this improved thermogenesis by revealing increased levels of sympathetic innervation markers, TH, and synaptophysin in the BAT. TH levels were also significantly elevated in the hypothalamus, a key thermoregulatory and metabolic center, suggesting neuronal GHSR deficiency enhanced sympathetic outflow from the central nervous system to the periphery, promoting thermogenesis. Collectively, these findings demonstrate that neuronal GHSR deficiency protects against age-associated thermogenic impairment, and this effect is likely mediated by increased sympathetic nervous system activity, leading to enhanced thermogenic programming in brown adipose tissue and subsequently improving insulin sensitivity in aging. These results suggest that targeting neuronal GHSR could be a promising therapeutic strategy to combat age-related metabolic dysregulation, such as thermogenic impairment and insulin resistance, as well as to prevent or treatment for cold-induced hypothermia.

Neuroinflammation is an integral part of inflammaging, linked to many age-associated dysfunctions and diseases [4]; thus, we evaluated brain inflammatory states in this study. We found that suppression of neuronal GHSR mitigates inflammation in the aging brain. Pro-inflammatory cytokines of IL-6 and IL-1β are usually low in the blood and tissues of young animals, but they are increased in chronic inflammatory conditions such as aging and obesity-associated metabolic diseases [37, 38]. A recent study revealed that suppressing IL-6 and IL-1β can alleviate brain aging and extend healthspan [39]. In our study, we found that suppression of neuronal GHSR reduces IL-6 and IL-1β levels in the brains of old mice. Cytokines are secreted by various cell types, including neurons [40]; while microglia and astrocytes are known to be major producers of these cytokines in the brain, neurons can also produce cytokines, particularly under stress. Aging can change the expression and regulation of these cytokines, potentially contributing to age-related neuroinflammation and cognitive decline [41–43]. We reported that inhibition of GHSR suppressed the palmitate-induced inflammatory response in Neuro-2A cells, evidenced by decreased IL-6, MCP1, and TNFα levels [15]. This finding aligns with the in vivo changes we observed in the brain of *Syn1‐cre;Ghsr *^*f/f*^ mice. Given this is a neuronal GHSR-deficient model, we believe that the decreased inflammation in the brain of the aging *Syn1‐cre Ghsr *^*f/f*^ mice, at least in part, is contributed by neurons. It would be important to study the role of microglia in GHSR-induced neuroinflammation, and we recently reported that GHSR deletion in a microglial cell line exhibited reduced inflammatory responses under fructose treatment [35]. The microglial-specific role of GHSR in neuroinflammation certainly warrants further investigation.

Aging is associated with cognitive functional decline [8]. It is well documented that cognitive function decreases with aging, along with the reduction of learning abilities [7]. In the novel object recognition test, old *Syn1‐cre;Ghsr*^*f/f*^ mice exhibited better recognition memory. Similarly, in the fear conditioning test, old *Syn1‐cre;Ghsr*^*f/f*^ mice demonstrated significantly better contextual and cued auditory memory. These results demonstrate that suppression of neuronal GHSR helps to preserve better learning and memory in aging. It is important to note that no significant differences were observed between young *Ghsr*^*f/f*^ mice and *Syn1‐cre;Ghsr *^*f/f*^ mice in any of these memory tests, suggesting that the benefits of neuronal GHSR suppression are associated with aging only.

BDNF is a critical neurotrophin essential for synaptic plasticity and cognition [36]. We demonstrated that neuronal GHSR ablation results in the upregulation of key neural plasticity-related markers in behaviorally relevant brain regions. Specifically, increased BDNF levels were observed in the cortex and hippocampus in old *Syn1‐cre;Ghsr *^*f/f*^ mice, while there was no difference between genotypes in young mice (data not shown). Moreover, there were increased synaptophysin levels in the cortex, DG, and CA3 areas of old *Syn1‐cre;Ghsr *^*f/f*^ mice, indicating enhanced synapse density, correlated with improved neural plasticity. Finally, increased TH expression in the cortex, amygdala, CA1, and CA3 further supports improved neuronal function and increased sympathetic activity. The sympathetic activity has been linked to enhanced thermogenesis and potentially cognitive benefits [44–46]. The increases in BDNF, synaptophysin, and TH collectively suggest neuronal GHSR deletion promotes neuronal plasticity and resilience in the aging brain.

While our studies have uncovered exciting insights related to the important roles of neuronal GHSR in metabolic and cognitive aging, we are aware that there are limitations in our study. We detected neural plasticity-relevant markers in the metabolic and cognitive-relevant brain regions. Electrophysiology could be more informative in studying the properties of excitatory and inhibitory postsynaptic potentials of neurons. All mice used in this study were male; no females were studied due to the extremely high housing cost of aging mice. Future studies in female mice would help determine whether the phenotypes we observed are relevant to both sexes. The sample size in some of our experiments was relatively small, as we lost some old mice near the end of their lifespan; we will increase the sample size accordingly in future aging studies. Inclusion of cell-specific cytokine expression data would help distinguish central from peripheral effects. We aim to improve all these caveats in future studies.

**Fig. 6:**
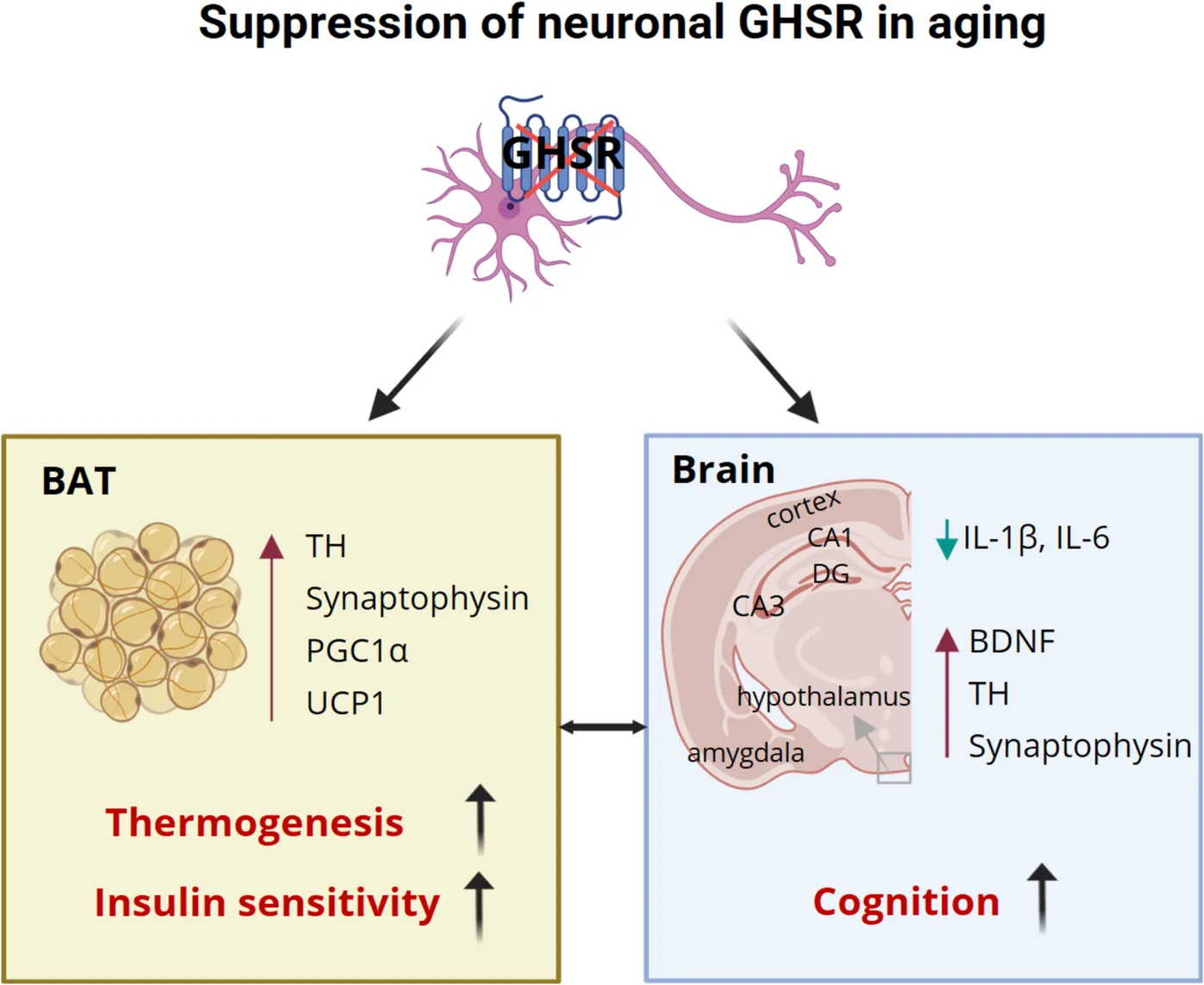
A schematic diagram illustrates the effects of neuronal GHSR ablation in old mice. In the BAT, the suppression of neuronal GHSR in aging increases thermogenic-related proteins (PGC1α, UCP1), and sympathetic innervation-related proteins (synaptophysin and TH); these changes together contribute to the improvement of thermogenesis under cold stress and insulin sensitivity. In the brain, the suppression of neuronal GHSR in aging reduces inflammation and increases neural plasticity-related proteins (BDNF, TH, and synaptophysin) in various brain regions; these changes collectively contribute to the improved metabolic and cognitive functions in aging. The schematic diagram was created with BioRender

In conclusion, it is the first investigation regarding the effect of neuronal GHSR suppression in aging-associated metabolic and cognitive decline. The findings strongly suggest that neuronal GHSR plays a significant role in age-related metabolic health and memory function. We found that neuronal GHSR suppression improved insulin sensitivity, glucose homeostasis, and thermogenesis in aging. We also found that suppression of neuronal GHSR protected against cognitive decline in aging, which is supported by decreased inflammatory cytokines and enhanced neural plasticity, as evidenced by the upregulation of BDNF, synaptophysin, and TH in key brain regions. Overall, this study provides proof-of-concept evidence that neuronal-specific GHSR suppression protects against age-associated metabolic and memory impairments by regulating thermogenesis and insulin sensitivity in the BAT, and modulating inflammation and neurotropic regulators in the brain (Fig. 6). These findings suggest that neuronal GHSR may serve as a promising therapeutic target for promoting healthy aging by combating age-related metabolic and cognitive dysfunctions.

## Supplementary Information

Below is the link to the electronic supplementary material.

ESM 1 BMP (12.9 MB)

ESM 2 BMP (17.8 MB)
